# Our New York Letter

**Published:** 1892-01

**Authors:** 


### Correspondence.

### NEW YORK LETTER.

NEW YORK, DEC. 21.

Dr. A. A. Smith, of this city, speaking recently on the subject of acute œdema of the lungs, said that the method of treatment he has found most successful in many cases is a combination of strychnine and atropine by hypodermic injection, and nitroglycerine by the mouth. The quantity used by him is 1-50 of a grain of strychnine and 1-100 of a grain of atropine given together, and 1-100 of a grain of nitroglycerine administered at the same time. The conditions that give rise to a pulmonary œdema, have also lessened the power of the stomach to take up medicinal agents. Nitroglycerine being so diffusible, is very readily absorbed and produces its effect very quickly and equally so when swallowed as when given subcutaneously. These doses are generally repeated in two hours. The strychnine, he says, increases the force of the heart by stimulation of its muscle, and its ganglia, as well as by stimulation of the vaso-motor centre. It is the most constant and powerful respiratory stimulant we possess. Atropine increases also the force of the heart and stimulates the vaso-motor and respiratory centres; while nitroglycerine dilates the arterial blood vessels, in this way relieving the venous conjestion. It relieves an overworked and weakened heart by its action on the arterial system, and affords an opportunity to strychnine and atropine to better produce their physiological effects.

After the first repetition, the atropine is discontinued and the nitroglycerine and strychnine are kept up every two hours until relief is afforded the patient.

Opium and nitroglycerine he has found to give the best results in pulmonary œdema occurring as a complication of acute renal disease. The sudden accumulation of poison acts on the nerve centres and on the vaso-motor nerves, interfering with their regulation of blood in the capillaries. Opium counteracts the effects of this poison, and nitroglycerine aids in the restoration of the functions of the capillaries. In acute so-called uremic poisoning, it is not at all uncommon, when the convulsions have been controlled, and the coma relieved by opium, to find the kidneys resuming their functions. The explanation of this phenomenon is not far to seek. The nerve supply of the kidneys has been acted upon in such a way by the accumulated poison as to destroy its functions temporarily. Opium neutralizes the effects of this poison on these nerves, affords them opportunity for rest, as it were, and they resume their normal function. The dose he usually gives is 1 6 of a grain of morphia and 1-100 of a grain of nitroglycerine, every two hours until relief is secured.

When cyanosis, with pulmonary œdema, is very marked, oxygen seemed to him to give relief to the dyspnœa, if cautiously administered. He has seen difficulty of breathing greatly aggravated where pure oxygen had been administered too freely. He has had more satisfactory results when it has been given with a tube, with the patient's mouth about two inches from the orifice, mixing it in this way with the atmospheric air. In fact a patient with pulmonary œdema will refuse to take a tube in his mouth because it obstructs his efforts at breathing.

In acute general pulmonary œdema, occurring in connection with pneumonia, strychnine, atropine, and nitroglycerine have frequently given relief.

In all operations upon the abdomen performed at the New York Hospital, the following general plan is carried out: For two or three days preceding the operation, the bowels are mildly purged and the diet carefully regulated. In some few cases, where the operation is long and the conditons favorable for shock, free stimulation by the mouth or rectum is resorted to.

The parts to be operated upon are carefully cleaned with soap, water, and ether, several hours previous to operation, and a large wet dressing of 1-2000 bichloride solution applied.

The instruments are always boiled for half an hour or more previous to the operation, and during the operation they are kept in a tray containing ether, boiled water or a 1-1000 solution of hydronaphthol. The sponges are kept in boiled water, and no antiseptics are used during the operation. The abdomen is irrigated only in those cases where there is some special indication. A small amount of 1-10,000 bichloride solution is used in a few cases where peritonitis is already advanced. The glass tube is the form of drainage generally used, but where there is any tendency to oozing from broken adhesions, a tampon of iodoform gauze is inserted alongside the tube and allowed to remain from twenty-four to forty-eight hours.

The peritoneum is sutured with a continuous catgut suture, the muscles with catgut interrupted sutures, and the skin by a separate line of interrupted silk sutures.

As soon as the patient is taken to the ward and there is any evidence of shock, the body is surrounded with hot water bottles, and hot water and whisky given by the rectum. During the first twenty-four hours, nothing is given by the mouth except a little hot water or cracked ice. Very little morphine is given in most cases. On the second day, liquid food in very small quantities is given. The drainage tubes are removed on the second or third day, and the sutures on the eighth or tenth. On the fourth day or earlier, if tympanites is present, the bowels are freely moved by small and frequently repeated doses of Rochelle salts.

Very little credit is to be placed in the sensational prophecies made in the newspapers regarding the occurrence of another epidemic of La Grippe in this city this winter. The history of epidemics of influenza shows that it does not repeat its visitations in any one locality, more frequently than every eight or ten years. The diagnosis of la grippe is not always an easy matter, and there is no evidence that the infection is as yet present in this city. The presence of la grippe is usually manifested by an increased mortality, and reports from the Health Board do not show such to be the case. The city has never been in a more healthful condition than it is at the present moment.

The following advertisement, which appeared in a recent issue of the New York Record, is full of suggestiveness for the young physicians who contemplate starting in practice in this city, and being a unique one of its kind is given verbatim.

"A young physician, practicing in this city for nearly two years, and earning less than one quarter of his expenses, desires to know of an opening where at least he can make a living."

Comment is unnecessary.

